# Dimensionless number is central to stress relaxation and expansive growth of the cell wall

**DOI:** 10.1038/s41598-017-03002-6

**Published:** 2017-06-07

**Authors:** Joseph K. E. Ortega

**Affiliations:** Bioengineering Laboratory, Department of Mechanical Engineering, University of Colorado Denver, 1200 Larimer Street, NC-2024-K, P.O. Box 173364, Denver, CO 80217-3364 USA

## Abstract

Experiments demonstrate that both plastic and elastic deformation of the cell wall are necessary for wall stress relaxation and expansive growth of walled cells. A biophysical equation (Augmented Growth Equation) was previously shown to accurately model the experimentally observed wall stress relaxation and expansive growth rate. Here, dimensional analysis is used to obtain a dimensionless Augmented Growth Equation with dimensionless coefficients (groups of variables, or Π parameters). It is shown that a single Π parameter controls the wall stress relaxation rate. The Π parameter represents the ratio of plastic and elastic deformation rates, and provides an explicit relationship between expansive growth rate and the wall’s mechanical properties. Values for Π are calculated for plant, algal, and fungal cells from previously reported experimental results. It is found that the Π values for each cell species are large and very different from each other. Expansive growth rates are calculated using the calculated Π values and are compared to those measured for plant and fungal cells during different growth conditions, after treatment with IAA, and in different developmental stages. The comparison shows good agreement and supports the claim that the Π parameter is central to expansive growth rate of walled cells.

## Introduction

Expansive growth is defined as a permanent increase in cell volume. Expansive growth of walled cells (e.g. algal, fungal and plant cells) depends on interrelated biochemical and physical processes. Active solutes inside their semi-permeable plasma membrane generate the osmotic potential needed to absorb water from its external environment and produce turgor pressure that stresses their walls. Wall stresses produce irreversible (plastic) and reversible (elastic) wall deformations in three orthogonal directions (volumetric wall deformation), generally stretching the wall in the two directions parallel to the wall surface (longitudinally and circumferentially) and contracting the wall in the direction perpendicular to the surface, making the wall thinner. New wall materials (polymers, proteins, etc.) are continually added to the inside surface to maintain a nearly constant wall thickness that varies between 0.1 μm and 1.0 μm, depending on the cell species.

Researchers have long recognized that plastic deformation of the cell wall is necessary for expansive growth and morphogenesis. It is generally thought that regulation of expansive growth rate of the cell wall chamber that encloses the cell is achieved by controlling the magnitude of plastic deformation of the wall and the magnitude of turgor pressure^1–4^. Using the constitutive equation (stress-strain relationship) for a Bingham fluid^5^, Lockhart^1^ derived a Growth Equation in terms of turgor pressure that describes these concepts, equation (1). Equation (1) describes the relative rate of change in volume of the cell wall chamber, *v*_cw_ = (d*V*/d*t*)/*V*, as equal to the relative volumetric plastic deformation rate of the cell wall chamber, *ϕ* (*P* − *P*_C_), when the turgor pressure, *P*, exceeds the critical turgor pressure, *P*_C_.

1 $${v}_{{\rm{cw}}}=\varphi (P-{P}_{{\rm{C}}})$$

The inclusive biophysical variables are *ϕ* (irreversible wall extensibility), *P* (turgor pressure), *P*_C_ (critical turgor pressure – a value that must be exceeded before plastic wall deformation occurs). It is noted that nearly all of the research conducted with this equation assumes that changes in volume occur predominately by changing the length of the cell, the diameter is assumed to remain relatively constant. The magnitude of *ϕ* has been shown to be biochemically controlled by the cell^6, 7^. Equation (1) explicitly indicates that expansive growth does not depend on elastic wall deformations, but only depends on plastic wall deformations and the inclusive biophysical variables (*ϕ*, *P*, and *P*_C_)^1, 3, 8^.

Equation (1) is simple, yet demonstrates broad utility in modeling and understanding expansive growth behavior of walled cells and is frequently found in many textbooks in plant biology and plant physiology. However, subsequent research demonstrates that equation (1) is not able to describe the results of experiments conducted specifically to reveal the mechanical behavior and constitutive relationship of growing cell walls. Specifically, equation (1) and its underlying constitutive equation for a Bingham fluid cannot describe the results of *in vivo* stress relaxation experiments^4, 9, 10^ and *in vivo* creep experiments that produce large changes in turgor pressure^11, 12^ because it does not describe reversible (elastic) wall deformations. Ortega^9^ used a constitutive equation derived in linear viscoelastic theory (Maxwell viscoelastic model) to accurately describe the results of stress relaxation experiments conducted on growing sporangiophores of the fungus, *Phycomyces blakesleeanus*. Subsequently, Ortega^13^ modified the Maxwell constitutive equation by replacing the Newtonian fluid inside the dashpot with a Bingham fluid (and later called it a Maxwell-Bingham viscoelastic model^6, 14^). The Maxwell-Bingham constitutive equation was used as the foundation for a biophysical wall extension equation in terms of turgor pressure, the Augmented Growth Equation^13^, equation (2).

2 $${v}_{{\rm{cw}}}=\varphi (P-{P}_{{\rm{C}}})+(\frac{1}{\varepsilon })\,\frac{{\rm{d}}P}{{\rm{d}}t}$$

The additional variables are *ε* (volumetric elastic modulus of the wall), and *t* (time). The Augmented Growth Equation describes the relative rate of change in volume of the cell wall chamber, *v*_cw_ = (d*V*/d*t*)/*V*, as the sum of the relative volumetric irreversible (plastic) deformation rate, *ϕ* (*P* − *P*_C_), and the relative volumetric reversible (elastic) deformation rate, (1/*ε*) d*P*/d*t*, of the cell wall chamber. Again, nearly all of the research conducted with this equation assumes that changes in volume occur predominately by changing the length of the cell, while the diameter is assumed to remain relatively constant. It is noted that equation (1) is recovered from equation (2) when the turgor pressure is constant. Importantly, the Augmented Growth Equation accurately describes the experimental results of *in vivo* stress relaxation experiments conducted on pea stems^10^ and fungal sporangiophores^4^, and *in vivo* creep experiments conducted on algal internode cells^11^ that involve large step changes in turgor pressure. The Augmented Growth Equation also demonstrates broad utility in modeling and understanding expansive growth behavior of walled cells^6, 14–16^, plant tissue^10, 17^ and whole plants^17, 18^.

Using the results of *in vivo* stress relaxation experiments, Cosgrove^10, 19, 20^ describes a mechanism for expansive growth of the cell wall that is more complicated than the simple wall extension mechanism modeled by Equation 1. Cosgrove^19^ states: “As a cell absorbs water, the wall extends passively, and polymers in the load-bearing network(s) are distended. In nongrowing cells, wall stress increases as the polymers are stretched like springs. Elastic energy is stored in the strained bonds of these polymers (and also in the increased order of the polymers), and this elastic energy does work on the cell protoplast by compressing it, thereby increasing its turgor pressure and water potential. When the cell water potential increases to the point where it matches that of the external water, net water uptake ceases. In growing cells, this equilibrium is never quite reached because the wall “relaxes,” which means that the load-bearing network breaks, slips, or is cut, and the distended polymers assume a more relaxed condition. Elastic energy of the wall is lost as heat, and a turgor reduction inevitably accompanies the reduced wall stress. Note, however, that this relaxation by itself does not entail a physical expansion of the wall or a change in cell volume. Turgor decreases because the wall simply stops compressing the protoplast. Expansion follows secondarily, as the cell absorbs water in response to the reduced water potential created by the reduction of turgor pressure”.

Interestingly, even though this “stress relaxation concept for expansive growth” has been available for a couple of decades, many of the conceptual models that are used to guide experimental investigations and mathematically models of expansive growth, still employ some form of equation (1), which cannot model stress relaxation. A few possible reasons for the continual employment of this concept and equation (1) are (i) it is simple to comprehend and to use, (ii) it is recovered from equation (2) when *P* is constant (thus elasticity is removed from the model when *P* is constant), and (iii) it is not apparent how to mathematically relate the stress-relaxation concept to expansive growth.

In this paper, dimensional analysis is used to obtain insight into the stress relaxation process and to explicitly demonstrate a relationship between expansive growth rate and wall elasticity as well as wall plasticity. Dimensional analysis is conducted on the Augmented Growth Equation, equation (2), describing plastic and elastic wall deformation rates. Three Π parameters are identified in the analysis, but only one is shown to control the turgor pressure decay rate that occurs during wall stress relaxation, the Π_pe_ parameter. Dimensional analysis provides a physical interpretation for the Π_pe_ parameter; it is the ratio of the relative volumetric *plastic* deformation rate of the wall and relative volumetric *elastic* deformation rate of the wall. This analytical result demonstrates that the *ratio* of plastic and elastic deformation rates are used to regulate the rate of stress relaxation that occurs during expansive growth of the cell wall chamber. It is shown that the relative magnitude of plastic and elastic deformation rates are important in controlling wall stress relaxation and expansive growth. Importantly, the Π_pe_ parameter provides a mathematical relationship between the relative rate of change in volume of the cell wall chamber, *v*_s_, and the mechanical properties of the wall, i.e. the irreversible wall extensibility, *ϕ*, and the volumetric elastic modulus, *ε*. It is noted that the turgor pressure, *P*, and critical turgor pressure, *P*_C_, do not appear in the Π_pe_ parameter. Values are calculated for the Π_pe_ parameter from previously reported experimental results obtained from cells in plants, fungi, and algae. It is found that the Π_pe_ values are large and very different for these different cell species. In some cases, data was available to calculate Π_pe_ values for the same cells when *ϕ* and *ε* changed. The results indicate that Π_pe_ can be used to calculate the magnitude of *v*_s_ when *ϕ* and *ε* change, because the calculated values for *v*_s_ compare very well with measured values for these cells. In addition, it is shown that the Π_pe_ parameter can be used to organize data related to cell wall deformation for *all* walled cells so that the ratio of plastic and elastic deformation rates of cell walls for different cells from plants, algae, and fungi can be compared.

## Theory, Analyses and Results

### Dimensional Analysis

The overall process of dimensional analysis consists of obtaining dimensionless groups of variables (Π parameters) involved in the process of interest and exploring the relationship between the Π parameters experimentally and/or analytically. A straightforward method to obtain the Π parameters can be employed when you have governing equations^21, 22^. In this method the variables within the governing equations are made dimensionless with appropriate constant reference quantities and the governing equations are manipulated to produce dimensionless equations and with dimensionless coefficients, or Π parameters. The Augmented Growth Equations represent governing equations for expansive growth^23^ and can be used for dimensional analyses^24^.

The expansive growth rate of a walled cell is determined by the *net* water uptake rate and cell wall deformation rate. During steady or quasi-steady growth, expansive growth rate is limited and controlled by the smaller rate. The magnitudes of net water uptake rate and wall deformation rate can be evaluated using the Π parameters identified in the dimensionless Augmented Growth Equations^24^. Scale analysis reveals that the net rate of water uptake is approximately eight times greater than the wall deformation rate for cells in growing pea stems (*Pisum satinis* L.), approximately 100 times greater for growing stage I sporangiophores (*P. blakesleeanus*), and approximately 12 times greater for growing stage IV sporangiophores (*P. blakesleeanus*); see ‘scale analysis’ in Calculations, Estimates, and Methods for details. These findings indicate that the smaller ‘wall deformation rate’ limits and controls the magnitude of expansive growth rate for these cells. Therefore, the analysis in this paper focuses on equation (2) that describes the wall deformation rate.

### Dimensionless Augmented Growth Equation

Here, dimensional analysis will focus on the Augmented Growth Equation that describes the wall deformation rate, equation (2). The analysis is conducted for walled cells growing normally at a quasi-steady rate^24^. Typically *P*_C_ is constant during normal growth and stress relaxation^6, 10, 12^, so *P*_C_ will be treated as a constant in the analysis. The variables (*v*_cw_, *t*, and *P*) in equation (2) are made dimensionless (*) with the following constant reference quantities^24^, *P*_C_ (critical turgor pressure) and *v*_s_ (steady or average relative volumetric growth rate, i.e. *v*_s_ = *v*_cw_ averaged over a time interval = constant).

$$\begin{array}{ccccccc}{P}^{\ast } & = & (\frac{P}{\,{P}_{{\rm{C}}}}) & {\rm{a}}{\rm{n}}{\rm{d}} & P & = & {P}^{\ast }\,{P}_{{\rm{C}}}\\ {t}^{\ast } & = & t{v}_{s} & {\rm{a}}{\rm{n}}{\rm{d}} & t & = & (\frac{{t}^{\ast }}{{v}_{s}\,})\\ {v}_{{\rm{c}}{\rm{w}}}^{\ast } & = & (\frac{{v}_{{\rm{c}}{\rm{w}}}}{\,{v}_{{\rm{s}}}}) & {\rm{a}}{\rm{n}}{\rm{d}} & {v}_{{\rm{c}}{\rm{w}}} & = & {v}_{{\rm{c}}w}^{\ast }{v}_{{\rm{s}}}\end{array}$$

Substituting the respective expressions that include the dimensionless variable and reference quantity (variables and their respective expressions in the right column above) for each of the variables into equation (2), we get equation (3):

3 $${v}_{{\rm{s}}}{v}_{{\rm{cw}}}^{\ast }=\varphi {P}_{{\rm{C}}}({P}^{\ast }-1)+(\frac{{v}_{{\rm{s}}}{P}_{{\rm{C}}}}{\varepsilon })\frac{{\rm{d}}{P}^{\ast }}{{\rm{d}}{t}^{\ast }}$$

The variables *v*_cw_*, P*, and t* are dimensionless. Therefore the dimensions, physical characteristics, and relative magnitude of each term are represented by their respective dimensional coefficient. Thus, *v*_s_ represents a steady or average magnitude of relative rate of change in volume of the cell wall chamber (constant), *ϕ P*_C_ represents the magnitude of the relative volumetric irreversible (plastic) deformation rate and (*v*_s_ *P*_C_/*ε*) represents the magnitude of the relative volumetric reversible (elastic) deformation rate. Dividing equation (3) by the constant reference quantity, *v*_s_, we get equation (4), the *dimensionless* Augmented Growth Equation^24^:

4 $${v}_{{\rm{cw}}}^{\ast }={{\rm{\Pi }}}_{{\rm{pv}}}({P}^{\ast }-1)+{{\rm{\Pi }}}_{{\rm{ev}}}\frac{{\rm{d}}{P}^{\ast }}{{\rm{d}}{t}^{\ast }\,}$$

The Π_pv_ and Π_ev_ parameters are dimensionless coefficients defined as follows^24^:

$${{\rm{\Pi }}}_{{\rm{pv}}}=(\frac{\varphi {P}_{{\rm{C}}}}{{v}_{{\rm{s}}}})=(\frac{relative\,volumetric\,plastic\,deformation\,rate\,of\,the\,wall}{relative\,volumetric\,growth\,rate}\,)$$

and

$${{\rm{\Pi }}}_{{\rm{ev}}}=(\frac{{P}_{{\rm{C}}}}{\varepsilon })=(\,\frac{relative\,volumetric\,elastic\,deformation\,rate\,of\,the\,wall}{relative\,volumetric\,growth\,rate}).$$

The Π parameters represents ratios of the dimensional coefficients shown in equation (3), where the first subscript of the Π parameter refers to the numerator and the second subscript refers to the denominator.

### Wall Stress Relaxation and Pressure Relaxation

Wall stress and pressure relaxation are the foundation of the “stress relaxation concept of expansive growth”^10, 19, 20^. Insight can be obtained by solving the dimensionless governing equation for stress relaxation. Typically, an *in vivo* stress relaxation experiment is conduct by isolating a growing walled cell from its water supply and eliminating the loss of water via transpiration. Then the decay in turgor pressure is measured as a function of time, usually with a pressure probe^4, 10^. It is essential to prevent water uptake and water loss from the cell (water volume and volume of the cell wall chamber must remain constant), so the decay in wall stress and concurrent decay in turgor pressure is the result of relaxation alone. Thus, experimentally and analytically, the condition that must be satisfied for a stress relaxation experiment is that the volume of the cell remains constant during the experiment, or *v*_cw_ = 0^10, 13^. Imposing the condition, *v*_cw_* = 0, the dimensionless governing equation for a stress relaxation experiment is obtained from equation (4):

$$0={{\rm{\Pi }}}_{{\rm{pv}}}({P}^{\ast }-1)+{{\rm{\Pi }}}_{{\rm{ev}}}\frac{{\rm{d}}{P}^{\ast }}{{\rm{d}}{t}^{\ast }\,}.$$

Rearranging, equation (5) is obtained where the dimensionless parameter, Π_pe_, is equal to the ratio, Π_pv_/Π_ev_:

5 $$\frac{{\rm{d}}{P}^{\ast }}{{\rm{d}}{t}^{\ast }}=-{{\rm{\Pi }}}_{{\rm{pe}}}({P}^{\ast }-1)$$

Using the dimensionless initial condition, P* = *P*_i_* = *P*_i_/*P*_C_, equation (5) is integrated and the dimensionless solution is obtained:

6 $${P}^{\ast }=({P}_{i}^{\ast }-1)\exp (-\,{{\rm{\Pi }}}_{{\rm{pe}}}{t}^{\ast })+1.$$

Where

7 $${{\rm{\Pi }}}_{{\rm{pe}}}=(\frac{\varepsilon \varphi }{{v}_{{\rm{s}}}})=(\frac{relative\,volumetric\,plastic\,deformation\,rate\,of\,the\,wall}{relative\,volumetric\,elastic\,deformation\,rate\,of\,the\,wall}).$$

Equation (5) demonstrates that the rate of decay of the dimensionless pressure, P*, during stress and pressure relaxation is directly related to the magnitude of Π_pe_. Equation (6) shows that the decay of the dimensionless pressure, P*, is exponential and the dimensionless time constant for the exponential decay is, *t*_c_* = (Π_pe_)^−1^. Equation (7) reveals that Π_pe_ is the ratio of relative volumetric plastic and elastic deformation rates of the wall.

### Magnitudes of Π_pe_ from *in vivo* stress relaxation and *in vivo* creep experiments

The magnitude of Π_pe_ can be calculated from results obtained from *in vivo* stress relaxation and *in vivo* creep experiments conducted with the pressure probe. In an *in vivo* stress relaxation experiment, the growing cell is removed from its water supply and prevented from transpiring^10, 13^. Usually this requires the tested cells to be incised from tissue^10^ or removed from mycelium (plucked)^4^. Then the exponential decay in *P* is measured with a pressure probe and the time constant for the exponential decay is, *t*_c_ = (*εϕ*)^−1^. The determination of Π_pe_, employing the *in vivo* stress relaxation method has the theoretical advantage that the product, *εϕ*, can be calculated directly from the halftime, T_1/2_, of the exponential decay of the turgor pressure; *εϕ* = *ln* 2/T_1/2_ and Π_pe_ = (*ln* 2/*v*_s_ T_1/2_).

**Figure 1:**
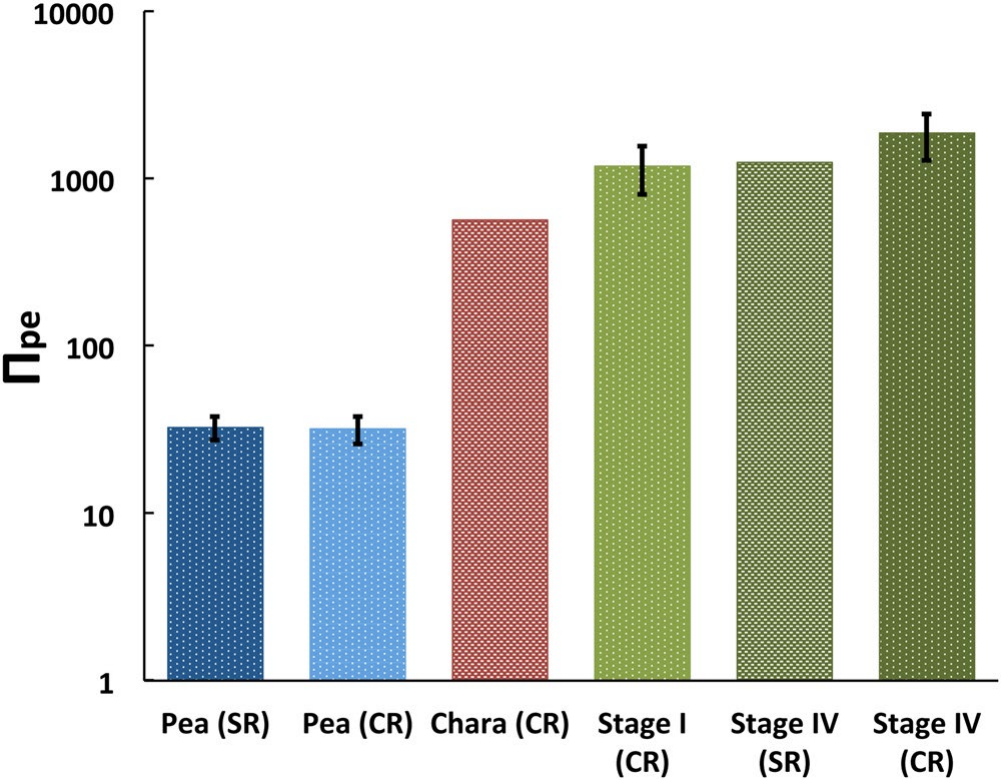
A comparison of the Π_pe_ values calculated for growing cells of pea stems (Pea)^10^, growing internode cells of *C. coralline* (Chara)^11, 12^, and growing sporangiophores of *P. blakesleeanus* (Stage I and Stage IV)^4, 6^. Note that the vertical scale for Π_pe_ is logarithmic. Also compared are the values obtained from *in vivo* stress relaxation (SR) and *in vivo* creep (CR) experiments for pea cells and stage IV sporangiophores. Only the *in vivo* creep experiments conducted on Stage I (CR) and Stage IV (CR) sporangiophores used *intact* cells (growing on the mycelium). The confidence intervals represent the maximum and minimum values of Π_pe_ that can be calculated from the statistical data (standard deviation and standard errors) presented in the respective papers^4, 6, 10^ (see Calculations, Estimates, and Methods for details).

Sometimes it is not possible to conduct *in vivo* stress relaxation experiments on walled cells. Then the magnitude of Π_pe_ can be calculated using equation (7) and the values of *ε*, *ϕ*, and *v*_s_ obtained from *in vivo* creep experiments^4, 6, 10, 11^. In an *in vivo* creep experiment the *P* in a growing cell is increased (stepped-up) with a pressure probe and the increase in elongation growth rate is measured. The magnitude of *ϕ* can be determined from the increase in elongation growth rate^4^. The magnitude of *ε* is determined separately from experiments during which a pulse-up in *P* is produced with the pressure probe^11^. Theoretically, *in vivo* creep and *in vivo* stress relaxation experiments should produce the same values for *ϕ* and (*εϕ*). Figure 1 compares Π_pe_ values determined from *in vivo* creep and *in vivo* stress relaxation experiments conducted on *incised* pea stem sections (*P. satinis* L.)^10^, *incised* algal internodes cells (*Chara corallina*)^11, 12^, *intact* stage I sporangiophores (*P. blakesleeanus*)^6^, *plucked* stage IV sporangiophores (*P. blakesleeanus*)^4^ and *intact* stage IV sporangiophores (*P. blakesleeanus*)^6^; see Calculations and Estimates for the calculations of Π_pe_ values. It should be noted that the vertical scale for Π_pe_ is logarithmic.

Figure 1 demonstrates that the Π_pe_ values for pea cells of *P. satinis* L., internode algal cells of *C. corallina*, and fungal sporangiophores of *P. blakesleeanus* are large and very different, by an order of magnitude or more. The different magnitudes of the Π_pe_ values presented in Fig. 1 provide a comparison of the ratio of plastic and elastic deformation rates of growing walls from plant, algal, and fungal cells.

### Expansive growth rates calculated from a constant Π_pe_

The steady or average relative volumetric growth rate, *v*_s_, can be calculated from Π_pe_ when *ε* and *ϕ* are measured. Rearranging equation (7) we get:

8 $${v}_{s}=\frac{\varepsilon \varphi \,}{{{\rm{\Pi }}}_{{\rm{p}}{\rm{e}}}}$$

Importantly, equation (8) provides an explicit mathematically relationship between expansive growth rate, *v*_s_, and stress relaxation of the cell wall, as characterized by Π_pe_. Also, equation (8) demonstrates that *v*_s_ can be determined without measuring *P* and *P*_C_, if Π_pe_ is known.

**Figure 2:**
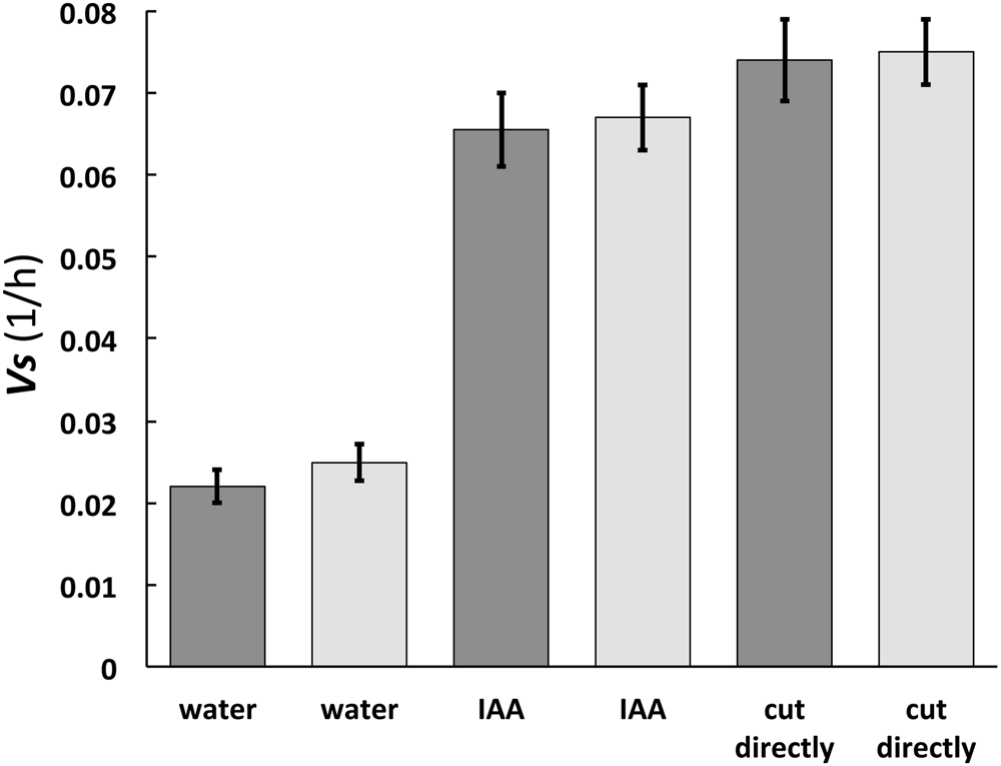
A comparison of *v*_s_ values calculated (dark grey) and measured (light grey) for cells in pea stems (*P. satinis* L.). Equation (9) is employed to calculate *v*_s_ values (dark grey) using a constant value for Π_pe_ (Π_pe_ = 32) and values for *ε* and *ϕ* taken from Cosgrove^10^; see Calculations, Estimates, and Methods for details. The confidence intervals for the measured values (light grey) are standard errors taken from Cosgrove^10^. The confidence intervals for the calculated values (dark grey) represents the maximum and minimum values that can be calculated from maximum and minimum values obtained from standard errors of *ϕ* ^10^.

**Figure 3:**
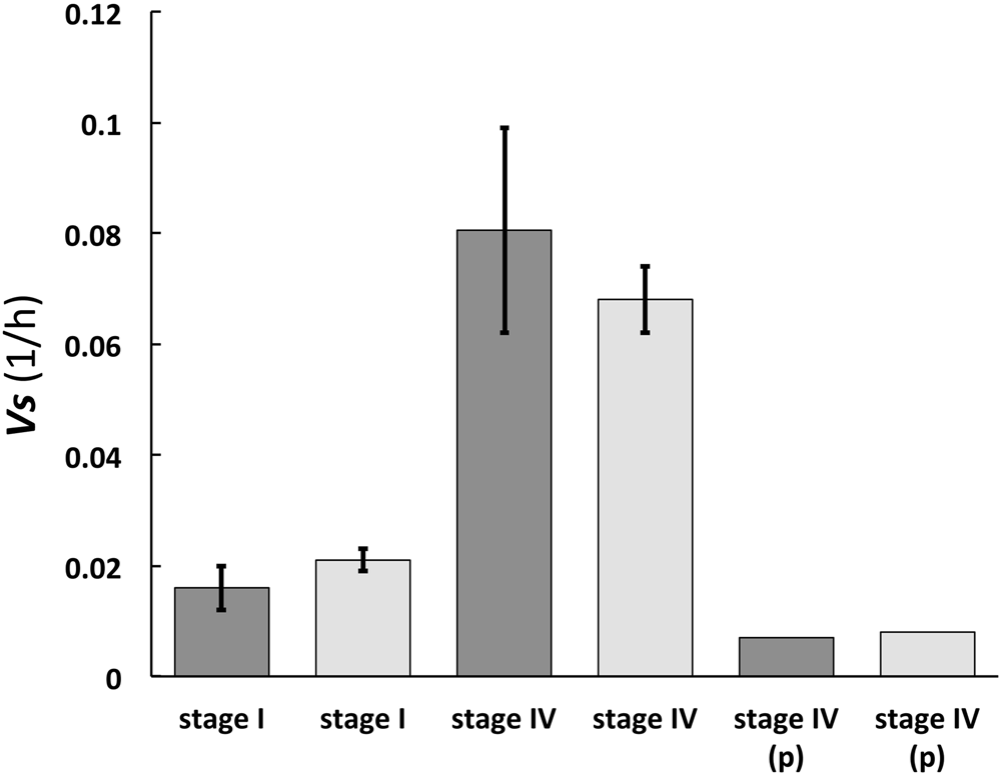
A comparison of *v*_s_ values calculated (dark grey) and measured (light grey) for stage I and stage IV sporangiophores of *P. blakesleeanus*. Equation (10) is employed to calculate *v*_s_ values (dark grey) using a constant value for Π_pe_ (Π_pe_ = 1524) and using values for *ε* and *ϕ* taken from Ortega *et al*.^4, 6^; see Calculations, Estimates, and Methods for details. The confidence intervals for the measured values (light grey) are standard errors taken from Ortega *et al*.^6^. The confidence intervals for the calculated values (dark grey) represents the maximum and minimum values that can be calculated from maximum and minimum values obtained from standard errors of *ε* and *ϕ* ^6^. The measured *v*_s_ value for the plucked (p) stage IV sporangiophore (light grey) is for a single experiment.

It is noted that the magnitude of Π_pe_ for pea cells of *P. satinis* L. calculated from *in vivo* stress relaxation experiments is the same as those calculated from *in vivo* creep experiments, even though the measured relative volumetric growth rates, *v*_sM_, of the cells during the *in vivo* creep experiments are much larger than for the *in vivo* stress relaxation experiments (~3 times larger). Based on maximum and minimum values of Π_pe_ that can be calculated from the statistical data, the magnitude of Π_pe_ for *intact* stage IV sporangiophores of *P. blakesleeanus* calculated from *in vivo* creep experiments is only slightly larger than the value calculated from *in vivo* stress relaxation experiments conducted on *plucked* stage IV sporangiophore, even though the measured relative volumetric growth rates, *v*_sM_, are much larger (~6 times larger). Again, based on maximum and minimum values of Π_pe_ that can be calculated from the statistical data, the magnitudes of Π_pe_ for stage IV and stage I sporangiophores are the same, even though the average value for *v*_sM_ of stage IV sporangiophores is more than four times larger than those of stage I sporangiophores. These results suggests that the ratios of plastic and elastic deformation rates (i.e. the magnitude of Π_pe_) of cell walls from a specific cell species are the same. It appears that the ratio of plastic and elastic deformation rates of cell walls from pea stems of *P. satinis* L. is 32 (i.e. Π_pe_ = 32). Similarly, it appears that the ratio of plastic and elastic deformation rates of cell walls from sporangiophores of *P. blakesleeanus* is approximately 1524 (i.e. Π_pe_ = 1524, the average value of intact stage I and stage IV from *in vivo* creep experiments). If this suggestion is correct, then a constant value for the Π_pe_ parameter can be used to calculate the steady relative volumetric growth rate, *v*_s_, for cells from pea stems of *P. satinis* L. and for sporangiophores of *P. blakesleeanus* when *ϕ* and/or *ε* change because of changes growth conditions (incised and growing in water and just cut from the plant), addition of growth hormone IAA, changes in development (stage I and stage IV), and intact versus plucked sporangiophores (see Figs 2 and 3). Specifically, *v*_s_ can be calculated for growing cells from pea stems by employing equation (9).

9 $${v}_{s}(P.satinis\,{\rm{L}}.)=\frac{\varepsilon \varphi }{32}$$

Equation (9) is employed to calculate *v*_s_ values using values for *ε* and *ϕ*, and compare them to measured values for pea stems of *P. satinis* L. (Fig. 2).

Equation (10) is employed to calculate *v*_s_ values using values for *ε* and *ϕ*, and compare them to measured values for stage I and stage IV sporangiophores of *P. blakesleeanus* (Fig. 3).

10 $${v}_{{\rm{s}}}(P.blakesleeanus)=\frac{\varepsilon \varphi }{1524}$$

## Discussion

Dimensional analysis reveals that the rate of wall stress relaxation is directly related to the magnitude of a single dimensionless parameter, Π_pe_; see equation (5). Equation (6) demonstrates that the dimensionless pressure (and stress) relaxation is exponential and the dimensionless time constant is, *t*_c_* = (Π_pe_)^−1^. Dimensional analysis provides a physical interpretation for the Π_pe_ parameter, it is the ratio of relative volumetric plastic and elastic deformation rates of the cell wall; see equation (7). The Π_pe_ parameter explicitly reveals a relationship between the steady or average relative volumetric growth rate of the cell, *v*_s_, and the wall stress relaxation as characterized by Π_pe_; see equation (7). In fact, *v*_s_ can be calculated using equation (8) when Π_pe_, *ϕ*, and *ε* are known; see equations (9) and (10). Equation (8) represents a method to calculate *v*_s_ when *P* or *P*_C_ are not known. Good agreement is obtained for calculated values of *v*_s_ compared to measured values, for cells from pea stems and sporangiophores (stage I and stage IV) when constant Π_pe_ values are used; see Figs 2 and 3.

Figure 1 compares magnitudes of Π_pe_ for cells from growing pea stems of *P. satinis* L. (Pea), growing internode cells of *C. coralline* (Chara), and growing sporangiophores of *P. blakesleeanus* (Stage I and Stage IV). The results indicate that the magnitude of Π_pe_ is very large (Π_pe_ ≫ 1) for all three species of walled cells. The large values for Π_pe_ indicate that the relative volumetric plastic wall deformation rate is very much larger than the relative volumetric elastic wall deformation rate during steady and quasi-steady expansive growth. From the perspective of a constitutive relationship (stress-strain relationship), the strain rate of the wall is proportional to the stress for plastic (irreversible) deformation, but proportional to the stress *rate* for elastic deformation. Therefore the constitutive relationship for plastic and elastic deformation are different. From an energy perspective, wall stress relaxation represents the dissipation of mechanical energy stored by elastic wall deformation. Plastic and elastic wall deformation rates represent the rates of two very different processes. An increase in elastic wall deformation rate represents an increase in the rate that mechanical energy is stored in the wall. This is a reversible process, and the stored mechanical energy can be recovered. An increase in plastic wall deformation rate represents an increase in rate of dissipation of stored mechanical energy in the wall. This is an irreversible process, and the lost mechanical energy cannot be recovered. The large values of Π_pe_ for the walled cells indicates that plastic wall deformation rate dominates wall deformation during steady and quasi-steady expansive growth. From the perspectives of constitutive relationships and energy, this finding draws into question the use of elastic models and elastic wall deformations to study expansive growth and morphogenesis.

Figure 1 also compares values for Π_pe_ determined from the results of *in vivo* creep and *in vivo* stress relaxation experiments. The results indicate that the Π_pe_ values are essentially identical for cells from pea stems and nearly statistically identical for stage IV sporangiophores. These experimental results are consistent with theoretical prediction that the Π_pe_ values obtained from *in vivo* creep and *in vivo* stress relaxation experiments should be the same. However, the results are also interesting because the growth rate for test specimens during *in vivo* stress relaxation experiments are three (for pea) to six (for stage IV) times smaller than during *in vivo* creep experiments. One wonders if the magnitude of Π_pe_ is invariant and is determined by a chemical wall loosening-hardening mechanism (chemorheology) that is fundamental to each species of cell, so that the same ratio of plastic and elastic wall deformation rates are produced for each cell species. If this suggestion were correct, it would make two predictions. First, it would predict that the chemorheology for cells in pea stems (*P. satinis* L.), algal internode cells (*C. corallina*) and sporangiophores (*P. blakesleeanus*) are different from each other because the magnitude of their respective values of Π_pe_ are very different from each other, by an order of magnitude or more. Second, it would predict that a single value for Π_pe_ represents a cell species and can be used for that cell species to calculate *v*_s_ when *ϕ* and/or *ε* change because of alterations in growth conditions, addition of growth hormone, and development.

There is evidence to support the first prediction. In a variety of higher plant cells within tissue, a pH-dependent protein (expansin) is found to loosen the wall by disrupting the hydrogen bonds between microfibrils^7, 25^. In large internode algal cells of *C. corallina*, experimental evidence indicates that making and breaking calcium bridges between pectin polymers loosens and hardens the wall^26, 27^. It is not known how the sporangiophore of *P. blakesleeanus* loosens and hardens its wall^28–30^, but it is predicted that its chemorheology will be different from those used by either cells in pea stems of *P. satinis* L. or internode algal cells of *C. coralline*, because the magnitude of Π_pe_ for the sporangiophores is orders of magnitude larger than those of either pea or Chara.

The second prediction was evaluated here by using a single value for Π_pe_ to calculate *v*_s_ for cells in pea stems (Π_pe_ = 32) and sporangiophores (Π_pe_ = 1524) when *ϕ* and/or *ε* change. Figure 2 compares calculated and measured *v*_s_ values for cells in pea stems (*P. satinis* L.) when *ϕ* changes because of changing growth conditions (‘incised and growing in water’ and ‘just cut from the plant’), and the addition of the growth hormone IAA. Figure 3 compares calculated and measured *v*_s_ values for the sporangiophores of *P. blakesleeanus* when *ϕ* and *ε* change because of development (stage I and stage IV) and when it is removed from the mycelium (plucked) and its base is put in pure water. The respective calculated and measured *v*_s_ values for cells in pea stems and sporangiophores compare very well and appear to be the statistically the same. It is concluded that the respective calculated and measured values of *v*_s_ for pea stems, stage IV, and stage I sporangiophores are the same. Therefore, it is also concluded that the results support the second prediction.

The results presented in Fig. 1 indicate that the magnitude of Π_pe_ for the sporangiophores of *P. blakesleeanus* is orders of magnitude larger, and significantly larger, than the magnitude of Π_pe_ for cells from pea stems. This finding indicates that wall deformation behavior can be significantly different for walled cells from different species. In the future, the Π_pe_ parameter can be determined for different species of walled cells. Then the Π_pe_ parameters can be used to provide insight into similarities and differences in the ratio of plastic and elastic wall deformation rates during expansive growth of different cell species. Future research may also provide more support for the idea that the magnitude of Π_pe_ is related to the chemorheology that regulates wall deformation when the wall is stressed. In future research, it would be interesting and important to determine Π_pe_ for ‘growth mutant’ cells^31^ and ‘wall mutant’ cells^32^, and compare their magnitudes to Π_pe_ values obtained from wild type cells of the same species. This could reveal whether the mutation produced a change in the ratio of plastic and elastic wall deformation rates.

The dimensionless parameters obtained and studied here are related to expansive growth rate and growth rate regulation. However, other studies concerning cell size and cell morphology have introduced different dimensionless parameters that include the elastic modulus of the wall, *E*, and the turgor pressure, *P*. Boudaoud^33^ introduced the dimensionless parameter, *E*/*P*, to study the size and shape of isolated walled cells. The cells are modeled as elastic shells where growth is driven by turgor pressure. Mechanical equilibrium is used to obtain scaling laws for cell size (cell radius) that employ the dimensionless parameter, *E*/*P*. The magnitude of the radius of the sporangiophores of *P. blakesleeanus* can generally be predicted with the published scaling equation. Goriely *et al*.^34^ introduced the dimensionless parameter, *P*_eff_ *w*/(*h E*), to characterize the overall wall deformation (*P*_eff_ is a measure of the normal stresses acting on the wall, *w* is width of the apical tip, and *h* is the thickness of the shell or wall). This research used nonlinear elastic models to get insight into how the shape of apical hyphae are produced. The general shape of apical hyphae produced with the dimensionless parameter is similar to those seen in stage I sporangiophores of *P. blakesleeanus*. Ortega^24^ obtained additional dimensionless Π parameters describing water uptake rates and transpiration rate (also see the ‘scale analysis’ in the Calculations, Estimates, and Methods) that may be used in future research to study expansive growth rate, water uptake rate, and transpiration rate of water-stressed cells in crop plants and may provide insight into how plants adapt to drought conditions. In general, different dimensionless parameters can be employed to obtain insight and to investigate different aspects of size, shape, and growth of walled cells.

**Figure 4:**
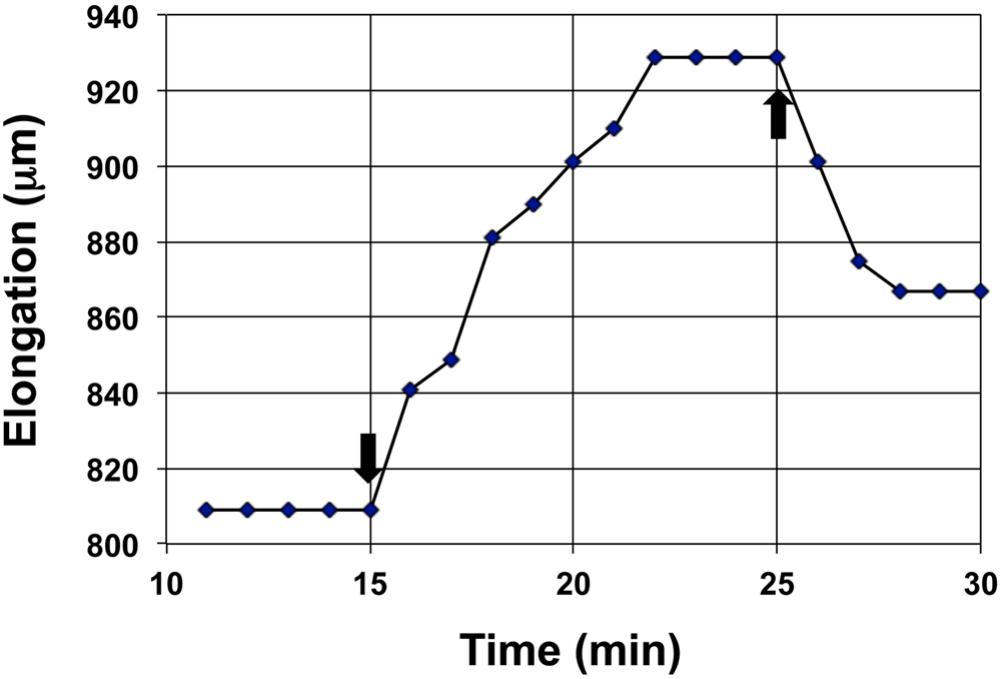
The results of a modified constant-tension extension experiment is presented. At ten minutes a weight (1.24 g) was used to apply constant tension to a five millimeter long section of a frozen-thawed wall that includes the growth zone^30^, and an initial extension of approximately 810 µm is observed (see Calculations, Estimates, and Methods – ‘Constant-tension extension experiment’ for more details). At 15 min (marked by the downward pointing arrow), 50 mM potassium-acetate buffer, pH = 4.5, replaces the bathing water (pH = 7.0) in the test apparatus^30^. Immediately, creep extension is observed until 22 min on the time scale. At 25 min (marked by the upward pointing arrow) the tension is released and the extension decreases (recovered elastic extension) until 28 min on the time scale.

In the physical sciences, Π parameters have been used to establish *similarity* between fluid flows, heat flows, and other transport processes by ensuring that the magnitudes of relevant Π parameters are the identical^21, 22, 35^. In the future, we plan to investigate the use of *wall deformation similarity* (by matching the magnitudes of the Π_pe_ parameter) to guide and valid *local* mathematical models that are making strides towards describing spatial and temporal wall deformation of the cell wall during expansive growth^36–42^. Also, future research will investigate the use of *wall deformation similarity* to augment experimental assays^30, 32^ that are used to investigate cell wall loosen-hardening mechanisms and chemorheology of cell walls. For example, constant-tension extension experiments were conducted on frozen and thawed walls of stage IV sporangiophore and showed that lowering the pH in the wall produces creep extension^30^. The creep extension is transient (~10 min in duration) and the creep extension rate is large for the first couple of minutes (~110 µm min^−1^), then decreases for the next eight minutes (~8 µm min^−1^) before it stops^30^. The pH mediated creep extension rates cover the range of the elongation rates exhibited by the stage IV sporangiophore (20–60 µm min^−1^). This result might suggest that lowering the pH in the living and growing cell wall is the mechanism employed by the sporangiophore to produce expansive growth of the wall and possibly regulate its growth rate. However, if the constant-tension extension protocol is modified so that the results can be used to calculate the magnitude of Π_pe_, then the magnitude of Π_pe_ for pH mediated creep extension can be compared to that obtained for natural growing stage IV sporangiophores. Figure 4 shows the results of a modified constant-tension extension experiment.

The magnitude of Π_pe_ is calculated from the experimental results (see Calculation, Estimates and Methods – ‘Constant-tension extension experiment’ for details). The calculated magnitude for Π_pe_ is 130 (Π_pe_ ≈ 130), which is an order of magnitude smaller than the magnitude obtained for normal growing stage IV sporangiophores (Π_pe_ ≈ 1500). This result suggests that decreasing the pH alone cannot account for the wall deformation rate that occurs in natural growing stage IV sporangiophores because it does not produce the same ratio of relative volumetric plastic and elastic deformation rate that occurs during normal growing stage IV sporangiophores. It is envisioned that the Π_pe_ parameter may be used in a similar way to assess the results of other experimental assays developed for higher plant cells^19, 32^.

## Calculations, Estimates, and Methods

### Scale analysis for ‘net water uptake rate’ and ‘wall deformation rate’

The following two *dimensionless* Augmented Growth Equations describe the net water uptake rate and the wall deformation rate^24^.

$${v}_{{\rm{w}}}^{\ast }={{\rm{\Pi }}}_{{\rm{wv}}}({\rm{\Delta }}{\pi }^{\ast }-{P}^{\ast })-{{\rm{\Pi }}}_{{\rm{Tv}}}{v}_{{\rm{T}}}^{\ast }(Net\,{\rm{of}}\,{\rm{water}}\,{\rm{uptake}}\,{\rm{rate}})$$

*Net water uptake rate* = *water uptake – water loss due to transpiration* (in relative terms)

$${v}_{{\rm{cw}}}^{\ast }={{\rm{\Pi }}}_{{\rm{pv}}}({P}^{\ast }-1)+{{\rm{\Pi }}}_{{\rm{ev}}}\frac{{\rm{d}}{P}^{\ast }}{{\rm{d}}{t}^{\ast }}({\rm{Wall}}\,{\rm{deformation}}\,{\rm{rate}})$$

*Wall deformation rate* = *plastic deformation* + *elastic deformation* (in relative terms)

The dimensionless Π parameters are defined as follows^24^:

$${{\rm{\Pi }}}_{{\rm{wv}}}=(\frac{L{P}_{{\rm{C}}}}{{v}_{{\rm{s}}}})=(\frac{{\rm{relative}}\,{\rm{volumetric}}\,{\rm{water}}\,{\rm{uptake}}\,{\rm{rate}}}{{\rm{relative}}\,{\rm{volumetric}}\,{\rm{growth}}\,{\rm{rate}}})$$

$${{\rm{\Pi }}}_{{\rm{Tv}}}=(\frac{{v}_{{\rm{sT}}}}{{v}_{{\rm{s}}}})=\,(\frac{{\rm{relative}}\,{\rm{volumetric}}\,{\rm{transpiration}}\,{\rm{rate}}}{{\rm{relative}}\,{\rm{volumetric}}\,{\rm{growth}}\,{\rm{rate}}})$$

$${{\rm{\Pi }}}_{{\rm{pv}}}=(\frac{\,\varphi {P}_{{\rm{C}}}}{{v}_{{\rm{s}}}})=(\frac{{\rm{relative}}\,{\rm{volumetric}}\,{\rm{plastic}}\,{\rm{deformation}}\,{\rm{rate}}\,{\rm{of}}\,{\rm{the}}\,{\rm{wall}}}{{\rm{relative}}\,{\rm{volumetric}}\,{\rm{growth}}\,{\rm{rate}}})$$

$${{\rm{\Pi }}}_{{\rm{ev}}}=(\frac{\,{P}_{{\rm{C}}}}{\varepsilon })=(\frac{{\rm{relative}}\,{\rm{volumetric}}\,{\rm{elastic}}\,{\rm{deformation}}\,{\rm{rate}}\,{\rm{of}}\,{\rm{the}}\,{\rm{wall}}}{{\rm{relative}}\,{\rm{volumetric}}\,{\rm{growth}}\,{\rm{rate}}})$$

The magnitude of each term in the dimensionless Augmented Growth Equations is determined by the magnitude of the dimensionless Π parameter associated with the term. So the magnitude of the ratio of net water uptake rate and wall deformation rate can be determine with the following Π_wd_ parameter:

$${{\rm{\Pi }}}_{{\rm{wd}}}=(\frac{{{\rm{\Pi }}}_{{\rm{wv}}}-{{\rm{\Pi }}}_{{\rm{Tv}}}}{{{\rm{\Pi }}}_{{\rm{pv}}}+{{\rm{\Pi }}}_{{\rm{ev}}}\,})$$

### Estimates of Π_wd_ for steady or quasi-steady growing cells in pea stem (*P. satinis* L.)

The values for the biophysical variables are obtained from Cosgrove^10^.

$${{\rm{\Pi }}}_{{\rm{wd}}}({\rm{pea}})=(\frac{\frac{L\,{P}_{{\rm{C}}}}{{v}_{{\rm{s}}}}-\,\frac{{v}_{{\rm{sT}}}}{{v}_{{\rm{s}}}}}{\frac{\varphi {P}_{{\rm{C}}}}{{v}_{{\rm{s}}}}+\frac{{P}_{{\rm{C}}}}{\varepsilon }\,})=\,(\frac{\frac{(\frac{2.0}{h\,MPa})(0.3MPa)}{\frac{0.0751}{h}}-0}{\frac{\,(\frac{0.25}{h\,MPa})(0.3MPa)}{\frac{0.0751}{h}}+\frac{\,(0.3MPa)}{(9.5MPa)}})\approx {\bf{7}}.{\bf{8}}$$

### Estimates of Π_wd_ for steady or quasi-steady growing sporangiophores (*P. blakesleeanus*)

The values for the biophysical variables are obtained from Ortega^6^, Cosgrove *et al*.^43^, and Ortega *et al*.^44^

$${{\rm{\Pi }}}_{{\rm{wd}}}({\rm{stage}}\,{\rm{I}})=(\frac{\frac{L{P}_{{\rm{C}}}}{{v}_{{\rm{s}}}}-\frac{{v}_{{\rm{sT}}}}{{v}_{{\rm{s}}}}}{\frac{\varphi {P}_{{\rm{C}}}}{{v}_{{\rm{s}}}}+\frac{\,{P}_{{\rm{C}}}}{\varepsilon }\,})=(\frac{\frac{(\frac{83.0}{h\,MPa})(0.4\,MPa)}{\frac{0.021}{h}}-\frac{0.12\,}{h\,}}{\frac{(\frac{0.81}{h\,MPa})(0.4MPa)}{\frac{0.021}{h}}+\frac{(0.4MPa)}{(69MPa)}})\approx {\bf{102}}$$

$${{\rm{\Pi }}}_{{\rm{wd}}}({\rm{stage}}\,{\rm{IV}})=(\frac{\frac{L{P}_{{\rm{C}}}}{{v}_{{\rm{s}}}}-\,\frac{{v}_{{\rm{sT}}}}{{v}_{{\rm{s}}}}}{\frac{\varphi {P}_{{\rm{C}}}}{{v}_{{\rm{s}}}}+\frac{{P}_{{\rm{C}}}}{\varepsilon }\,})=(\frac{\frac{(\frac{23.5}{h\,MPa})(0.26MPa)}{\frac{0.068}{h}}-\frac{0.12}{h}}{\frac{(\frac{2.0}{h\,MPa})(0.26MPa)}{\frac{0.068}{h}}+\frac{(0.26MPa)}{(61MPa)}})\approx {\bf{11.7}}$$

### Estimates for Π_pe_ from *in vivo* stress relaxation experiments

The magnitude of Π_pe_ = *ln* 2/(*v*_s_ T_1/2_) is estimated for pea stems and fungal sporangiophores. The following data was obtained from *incised* pea stems sections (*P. satinis* L.) that include the growth zone^10^ (for Fig. 1). The stems sections were incised so that they can be removed from their water supply and transpiration eliminated:

$$\begin{array}{rcl}{{\boldsymbol{v}}}_{{\bf{s}}} & = & {\bf{0.0249}}{\boldsymbol{\pm }}{\bf{0.0022}}\,({\bf{SE}})\,{{\boldsymbol{h}}}^{-{\bf{1}}}\\ {v}_{s}({\rm{largest}}) & = & (0.0249+0.0022)\,{h}^{-1}=0.0271\,{h}^{-1}\\ {v}_{s}({\rm{smallest}}) & = & (0.0249-0.0022)\,{h}^{-1}=0.0227\,{h}^{-1}\\ {{\bf{T}}}_{{\bf{1}}/{\bf{2}}} & = & {\bf{52.2}}\pm {\bf{3.7}}\,({\bf{SE}})\,{\bf{\min }}={\bf{0.87}}\pm {\bf{0.06}}\,({\bf{SE}})\,{{\bf{h}}}^{-1}\\ \varepsilon \varphi ({\rm{largest}}) & = & ln2/{T}_{1/2}=ln2/0.81\,h=0.86\,{{\rm{h}}}^{-1}\\ \varepsilon \varphi ({\rm{smallest}}) & = & ln2/{T}_{1/2}=ln2/0.93\,h=0.75\,{{\rm{h}}}^{-1}\\ {{\boldsymbol{\Pi }}}_{{\bf{pe}}}({\rm{largest}}) & = & (\frac{\varepsilon \varphi ({\rm{largest}})}{{v}_{{\rm{s}}}({\rm{smallest}})})=(\frac{(0.8557)\,{h}^{-1}}{(0.0227\,{h}^{-1})})\cong {\bf{37}}.{\bf{7}}\\ {{\boldsymbol{\Pi }}}_{{\bf{pe}}}({\rm{smallest}}) & = & (\frac{\varepsilon \varphi ({\rm{smallest}})}{{v}_{{\rm{s}}}({\rm{largest}})})=(\frac{(0.7453)\,{h}^{-1}}{(0.0271\,{h}^{-1})})\cong {\bf{27}}.{\bf{5}}\\ {{\boldsymbol{\Pi }}}_{{\bf{pe}}}({\bf{pea}}) & = & {\bf{32.6}}\pm {\bf{5.1}}\end{array}$$

The following data was obtained for *plucked* stage IV sporangiophores of *P. blakesleeanus* ^4^. The sporangiophores were plucked (carefully removed from the mycelium) so that they could be put in an environmental chamber where the water is removed and the air is maintained at 100% RH to eliminate transpiration.

$$\begin{array}{rcl}l & = & 40\,mm=4\times {10}^{4}\,\mu m\\ \frac{{\rm{d}}l}{{\rm{d}}t} & = & 5.5\,\mu m\,mi{n}^{-1}=330\,\mu m\,{h}^{-1}\\ {v}_{{\rm{s}}} & = & (\frac{{\rm{d}}l}{l\,{\rm{d}}t})=0.0083\,{h}^{-1}\\ {{\rm{T}}}_{1/2} & = & 4\,min=0.067\,h\\ \varepsilon \varphi  & = & (\frac{ln2}{{{\rm{T}}}_{1/2}})=(\frac{ln2}{0.067\,h})=10.3\,{h}^{-1}\\ {{\boldsymbol{\Pi }}}_{{\bf{pe}}} & = & (\frac{\varepsilon \varphi }{{v}_{{\rm{s}}}})=(\frac{ln2}{{v}_{{\rm{s}}}{{\rm{T}}}_{1/2}})=(\frac{(0.693)\,}{(0.0083\,{h}^{-1})(0.067\,h)})\cong {\bf{1246}}\end{array}$$

### Estimates for Π_pe_ from *in vivo* creep experiments

The magnitude of Π_pe_ = (*εϕ*/*v*_s_) is estimated for pea stems, internode algal cells, and fungal sporangiophores (for Fig. 1). The following values were obtained for *incised* pea stems of *Pisum satinis* L.^10^:

$$\begin{array}{rcl}{{\boldsymbol{v}}}_{{\bf{s}}} & = & (\frac{{\bf{d}}{\boldsymbol{l}}}{{\boldsymbol{l}}\,{\bf{d}}{\boldsymbol{t}}})={\bf{0.0751}}{\boldsymbol{\pm }}{\bf{0.0043}}\,({\boldsymbol{SE}})\,{{\boldsymbol{h}}}^{-{\bf{1}}}\\ {v}_{{\rm{s}}}({\rm{largest}}) & = & (0.0751+0.0043)\,{h}^{-1}=0.0794\,{h}^{-1}\\ {v}_{{\rm{s}}}({\rm{smallest}}) & = & (0.0751-0.0043)\,{h}^{-1}=0.0667\,{h}^{-1}\\ {\boldsymbol{\varphi }} & = & {\bf{0.25}}\pm {\bf{0.017}}\,{{\boldsymbol{h}}}^{-{\bf{1}}}{\boldsymbol{MP}}{{\boldsymbol{a}}}^{-{\bf{1}}}\\ \varphi ({\rm{largest}}) & = & 0.25+\,0.017{h}^{-1}MP{a}^{-1}=0.267\\ \varphi ({\rm{smallest}}) & = & 0.25-0.017\,{h}^{-1}MP{a}^{-1}=0.233\\ \varepsilon  & = & 9.5\,MPa\\ {{\boldsymbol{\Pi }}}_{{\bf{pe}}}({\rm{mean}}) & = & (\frac{\varepsilon \varphi }{{v}_{{\rm{s}}}})=(\frac{(9.5\,MPa)(0.25\,{h}^{-1}MP{a}^{-1})}{(0.0751\,{h}^{-1})})\approx {\bf{31.6}}\\ {{\boldsymbol{\Pi }}}_{{\bf{pe}}}({\rm{largest}}) & = & (\frac{\,\varepsilon \varphi ({\rm{largest}})}{{v}_{{\rm{s}}}({\rm{smallest}})})=(\frac{(9.5\,MPa)(0.267\,{h}^{-1}MP{a}^{-1})}{(0.0667\,{h}^{-1})})\approx {\bf{38}}\\ {{\boldsymbol{\Pi }}}_{{\bf{pe}}}({\rm{smallest}}) & = & (\frac{\varepsilon \varphi ({\rm{smallest}})}{{v}_{{\rm{s}}}({\rm{largest}})})\,=(\frac{(9.5\,MPa)(0.233\,{h}^{-1}MP{a}^{-1})}{(0.0794\,{h}^{-1})})\approx {\bf{27}}.{\bf{9}}\\ {{\boldsymbol{\Pi }}}_{{\bf{pe}}}({\bf{pea}}) & \approx  & {\bf{32}}\pm {\bf{6}}\end{array}$$

The following values were used for incised internode algal cells of *C. corallina* ^11, 12^:

$$\begin{array}{rcl}l & = & 13\,mm=1.3\times {10}^{4}\,\mu m\\ \frac{{\rm{d}}l}{{\rm{d}}t} & = & 0.014\,\mu m{s}^{-1}=50.4\,\mu m\,{h}^{-1}\\ {v}_{{\rm{s}}} & = & (\frac{{\rm{d}}l}{l\,{\rm{d}}t})=1.1\times {10}^{-6}\,{s}^{-1}=0.0039\,{h}^{-1}\\ m & = & (\frac{{\rm{d}}l}{(P-{P}_{{\rm{C}}}){\rm{d}}t})=(\frac{50.4\,\mu m\,{h}^{-1}}{(0.527-0.35)\,MPa})=285\,\mu m{h}^{-1}MP{a}^{-1}\\ \varphi  & = & (\frac{m}{l})=(\frac{285\,\mu m\,\,{h}^{-1}MP{a}^{-1}}{1.3\times {10}^{4}\,\mu m})=0.022\,{h}^{-1}MP{a}^{-1}\\ \varepsilon  & = & 100\,MPa\\ {{\boldsymbol{\Pi }}}_{{\bf{pe}}} & = & (\frac{\varepsilon \varphi }{{v}_{{\rm{s}}}})=(\frac{(100\,MPa)(0.022\,{h}^{-1}MP{a}^{-1})}{(0.0039\,{h}^{-1})})\cong {\bf{564}}\end{array}$$

The following values were obtained for *intact* stage IV sporangiophores of *Phycomyces blakesleeanus* ^4, 6^:

$$\begin{array}{rcl}l & = & 30\,mm=3.0\times {10}^{4}\,\mu m\\ \frac{{\bf{d}}{\boldsymbol{l}}}{{\bf{d}}{\boldsymbol{t}}} & = & {\bf{34}}\pm {\bf{3.1}}\,({\bf{SE}})\,{\boldsymbol{\mu m}}\,{\boldsymbol{mi}}{{\boldsymbol{n}}}^{-1}({\bf{n}}={\bf{20}})\,\\ {v}_{{\rm{s}}}({\rm{largest}}) & = & (\frac{{\rm{d}}l}{l\,{\rm{d}}t})=(\frac{34.1+3.1\,\mu {\rm{m}}/{\rm{\min }}}{30000\mu m})=0.0012\,mi{n}^{-1}=0.074\,{h}^{-1}\\ {v}_{{\rm{s}}}({\rm{smallest}}) & = & (\frac{{\rm{d}}l}{l\,{\rm{d}}t})=(\frac{34.-3.1\,\mu {\rm{m}}/{\rm{\min }}}{\,30000\,\mu m})=0.0010\,mi{n}^{-1}=0.062\,{h}^{-1}\\ {\boldsymbol{m}} & = & {\bf{997}}\pm {\bf{160}}\,({\bf{SE}})\,{\boldsymbol{\mu }}{\bf{m}}\,{\boldsymbol{mi}}{{\boldsymbol{n}}}^{-1}{\boldsymbol{MP}}{{\boldsymbol{a}}}^{-1}({\boldsymbol{n}}={\bf{20}})\\ \varphi ({\rm{largest}}) & = & (\frac{m}{l})=(\frac{997+160\,\mu m\,mi{n}^{-1}MP{a}^{-1}}{\,\,3.0\times {10}^{4}\,\mu m})\\  & = & 0.039\,mi{n}^{-1}MP{a}^{-1}=2.3\,{h}^{-1}MP{a}^{-1}\\ \varphi ({\rm{smallest}}) & = & (\frac{m}{l})=(\frac{997-160\,\mu m\,mi{n}^{-1}MP{a}^{-1}}{3.0\times {10}^{4}\,\mu m})\\  & = & 0.028\,mi{n}^{-1}MP{a}^{-1}=1.7\,{h}^{-1}MP{a}^{-1}\\ {\boldsymbol{\varepsilon }} & = & {\bf{60.9}}\pm {\bf{5.1}}\,({\bf{S}}{\bf{E}})\,{\boldsymbol{MPa}}({\boldsymbol{n}}={\bf{27}})\\ {{\boldsymbol{\Pi }}}_{{\bf{pe}}}({\rm{largest}}) & = & (\frac{\varepsilon \varphi ({\rm{largest}})}{{v}_{{\rm{s}}}({\rm{smallest}})})=(\frac{(66\,MPa)(2.3\,{h}^{-1}MP{a}^{-1})}{(0.062\,{h}^{-1})})\approx {\bf{2448}}\\ {{\boldsymbol{\Pi }}}_{{\bf{pe}}}({\rm{smallest}}) & = & (\frac{\varepsilon \varphi \,({\rm{smallest}})}{{v}_{{\rm{s}}}({\rm{largest}})})\,=(\frac{(55.8\,MPa)(1.7\,{h}^{-1}MP{a}^{-1})\,}{(0.074\,{h}^{-1})})\approx {\bf{1282}}\\ {{\boldsymbol{\Pi }}}_{{\bf{pe}}}({\bf{stage}}\,{\bf{IV}}) & = & {\bf{1865}}\pm {\bf{583}}\end{array}$$

The following values were obtained for *intact* stage I sporangiophores of *Phycomyces blakesleeanus* ^6^:

$$\begin{array}{rcl}l & = & 20\,mm=2.0\times {10}^{4}\,\mu m\\ \frac{{\bf{d}}{\boldsymbol{l}}}{{\bf{d}}{\boldsymbol{t}}} & = & {\bf{7.1}}\pm {\bf{0.7}}\,({\bf{SE}})\,{\boldsymbol{\mu m}}\,{\boldsymbol{mi}}{{\boldsymbol{n}}}^{-{\bf{1}}}({\bf{n}}={\bf{17}})\\ {v}_{{\rm{s}}}({\rm{largest}}) & = & (\frac{{\rm{d}}l}{l\,{\rm{d}}t})=(\frac{7.1+0.7\,\mu {\rm{m}}/{\rm{\min }}}{\,20000\,\mu m})=0.00039\,mi{n}^{-1}=0.023\,{h}^{-1}\\ {v}_{{\rm{s}}}({\rm{smallest}}) & = & (\frac{{\rm{d}}l}{l\,{\rm{d}}t})=(\frac{7.1\,-0.7\,\mu {\rm{m}}/{\rm{\min }}}{\,20000\,\mu m})=0.00032\,mi{n}^{-1}=0.019\,{h}^{-1}\\ {\boldsymbol{m}} & = & {\bf{116}}\pm {\bf{18.8}}\,({\bf{SE}})\,{\boldsymbol{\mu }}{\boldsymbol{m}}\,{\boldsymbol{mi}}{{\boldsymbol{n}}}^{-{\bf{1}}}{\boldsymbol{MP}}{{\boldsymbol{a}}}^{-{\bf{1}}}({\boldsymbol{n}}={\bf{17}})\\ \varphi ({\rm{largest}}) & = & (\frac{m}{l})=(\frac{116+18.8\,\mu m\,mi{n}^{-1}MP{a}^{-1}}{2.0\times {10}^{4}\mu m})\\  & = & 0.00674\,mi{n}^{-1}MP{a}^{-1}=0.4044\,{h}^{-1}MP{a}^{-1}\\ \varphi ({\rm{smallest}}) & = & (\frac{m}{l})=(\frac{116-18.8\,\mu m\,mi{n}^{-1}MP{a}^{-1}}{2.0\times {10}^{4}\mu m})\\  & = & 0.00486\,mi{n}^{-1}MP{a}^{-1}=0.29\,{h}^{-1}MP{a}^{-1}\\ {\boldsymbol{\varepsilon }} & = & {\bf{68.9}}\pm {\bf{5.6}}\,({\bf{SE}})\,{\boldsymbol{MPa}}\,({\boldsymbol{n}}={\bf{27}})\\ {{\boldsymbol{\Pi }}}_{{\bf{pe}}}(largest) & = & (\frac{\varepsilon \varphi (largest)\,}{{v}_{{\rm{s}}}(smallest)})=(\frac{(74.5\,MPa)(0.40\,{h}^{-1}MP{a}^{-1})}{(0.019\,{h}^{-1})})\approx {\bf{1568}}\\ {{\boldsymbol{\Pi }}}_{{\bf{pe}}}(smallest) & = & (\frac{\varepsilon \varphi (smallest)}{{v}_{{\rm{s}}}(l\text{arg}est)})=(\frac{(63.3\,MPa)(0.29\,{h}^{-1}MP{a}^{-1})\,}{(0.023\,{h}^{-1})})\approx {\bf{798}}\\ {{\boldsymbol{\Pi }}}_{{\bf{pe}}}({\bf{stage}}\,{\bf{I}}) & = & {\bf{1183}}\pm {\bf{385}}\,\end{array}$$

### *v*_s_ values for cells from pea stems using Π_pe_ = 32

This data for pea stems was taken directly from Cosgrove^10^, for data labeled “Water” (for Fig. 2). The value for *ε* is located in the text. ***v***_**sM**_ is the measured relative volumetric growth rate and ***v***_**sC**_ is the calculated relative volumetric growth rate.

$$\begin{array}{rcl}{{\boldsymbol{v}}}_{{\bf{sM}}} & = & {\bf{0.0249}}\pm {\bf{0.0022}}\,({\bf{SE}})\,{{\boldsymbol{h}}}^{-{\bf{1}}}\,({\bf{n}}={\bf{10}})\\ \varepsilon  & = & 9.5\,MPa\\ {\boldsymbol{\varphi }} & = & {\bf{0.074}}\pm {\bf{0.007}}{{\boldsymbol{h}}}^{-1}{\boldsymbol{MP}}{{\boldsymbol{a}}}^{-{\bf{1}}}\\ {{\boldsymbol{v}}}_{{\bf{sC}}}({\rm{largest}}) & = & (\frac{{\boldsymbol{\varepsilon }}{\boldsymbol{\varphi }}}{{{\boldsymbol{\Pi }}}_{{\bf{pe}}}})=(\frac{(9.5\,MPa\,)(0.074+0.007\,MP{a}^{-1}{h}^{-1})}{32})\cong {\bf{0.024}}{{\boldsymbol{h}}}^{-{\bf{1}}}\\ {{\boldsymbol{v}}}_{{\bf{sC}}}({\rm{smallest}}) & = & (\frac{{\boldsymbol{\varepsilon }}{\boldsymbol{\varphi }}}{{{\boldsymbol{\Pi }}}_{{\bf{pe}}}})\,=(\frac{(9.5\,MPa\,)(0.074-0.007\,MP{a}^{-1}{h}^{-1})}{32})\cong {\bf{0.020}}{{\boldsymbol{h}}}^{-1}\\ {{\boldsymbol{v}}}_{{\bf{sC}}} & = & {\bf{0.022}}\pm {\bf{0.002}}\,{{\boldsymbol{h}}}^{-{\bf{1}}}\end{array}$$

This data for pea stems was taken directly from Cosgrove^10^, for data labeled “IAA”. The value for ε that is located in the text.

$$\begin{array}{rcl}{{\boldsymbol{v}}}_{{\bf{sM}}} & = & {\bf{0.067}}\pm {\bf{0.004}}\,({\bf{SE}})\,{{\boldsymbol{h}}}^{-{\bf{1}}}\,({\bf{n}}={\bf{8}})\\ \varepsilon  & = & 9.5\,MPa\\ {\boldsymbol{\varphi }} & = & {\bf{0.22}}\pm {\bf{0.014}}\,{{\boldsymbol{h}}}^{-{\bf{1}}}{\boldsymbol{MP}}{{\boldsymbol{a}}}^{-{\bf{1}}}\\ {{\boldsymbol{v}}}_{{\bf{sC}}}({\rm{largest}}) & = & (\frac{{\boldsymbol{\varepsilon }}{\boldsymbol{\varphi }}}{{{\boldsymbol{\Pi }}}_{{\bf{pe}}}})=(\frac{(9.5\,MPa)(0.22+0.014\,MP{a}^{-1}{h}^{-1})}{32})\cong {\bf{0.070}}{{\boldsymbol{h}}}^{-1}\\ {{\boldsymbol{v}}}_{{\bf{sC}}}({\rm{smallest}}) & = & (\frac{{\boldsymbol{\varepsilon }}{\boldsymbol{\varphi }}}{{{\boldsymbol{\Pi }}}_{{\bf{pe}}}})=(\frac{(9.5\,MPa)(0.22-0.0147\,MP{a}^{-1}{h}^{-1})}{32})\cong {\bf{0.061}}{{\boldsymbol{h}}}^{-{\bf{1}}}\\ {{\boldsymbol{v}}}_{{\bf{sC}}} & = & {\bf{0.0655}}\pm {\bf{0.0045}}{{\boldsymbol{h}}}^{-{\bf{1}}}\end{array}$$

This data for pea stems was taken directly from Cosgrove^10^, for data labeled “Cut directly form plant”. The value for *ε* is located in the text.

$$\begin{array}{rcl}{{\boldsymbol{v}}}_{{\bf{sM}}} & = & {\bf{0.075}}\pm {\bf{0.004}}\,({\bf{SE}})\,{{\boldsymbol{h}}}^{-{\bf{1}}}({\bf{n}}={\bf{10}})\\ \varepsilon  & = & 9.5MPa\\ {\boldsymbol{\varphi }} & = & {\bf{0.25}}\pm {\bf{0.017}}{{\boldsymbol{h}}}^{-{\bf{1}}}{\boldsymbol{MP}}{{\boldsymbol{a}}}^{-{\bf{1}}}\\ {{\boldsymbol{v}}}_{{\bf{sC}}}({\rm{largest}}) & = & (\frac{{\boldsymbol{\varepsilon }}{\boldsymbol{\varphi }}}{{{\boldsymbol{\Pi }}}_{{\bf{pe}}}})=(\frac{(9.5\,MPa\,)(0.25+0.017MP{a}^{-1}{h}^{-1})}{32})\cong {\bf{0.079}}{{\boldsymbol{h}}}^{-{\bf{1}}}\\ {{\boldsymbol{v}}}_{{\bf{sC}}}({\rm{smallest}}) & = & (\frac{{\boldsymbol{\varepsilon }}{\boldsymbol{\varphi }}}{{{\boldsymbol{\Pi }}}_{{\bf{pe}}}})=(\frac{(9.5\,MPa)(0.25-0.017MP{a}^{-1}{h}^{-1})}{32})\cong {\bf{0.069}}{{\boldsymbol{h}}}^{-{\bf{1}}}\\ {{\boldsymbol{v}}}_{{\bf{sC}}} & = & {\bf{0.074}}\pm {\bf{0.005}}{{\boldsymbol{h}}}^{-{\bf{1}}}\end{array}$$

### *v*_sC_ values for sporangiophores using Π_pe_ = 1524

The data for *plucked* stage IV sporangiophore was taken from Ortega *et al*.^4^ and Ortega^6^. ***v***_**sM**_ is the measured relative volumetric growth rate and ***v***_**sC**_ is the calculated relative volumetric growth rate (for Fig. 3). For ***v***_**sM**_, d*L*/d*t* is measured and then divided by *l*.

$$\begin{array}{rcl}l & = & 40\,mm=4\times {10}^{4}\,\mu m\\ \frac{{\rm{d}}l}{{\rm{d}}t} & = & 5.5\,\mu m\,mi{n}^{-1}=330\,\mu m\,{h}^{-1}\\ {{\boldsymbol{v}}}_{{\bf{sM}}} & = & (\frac{{\rm{d}}l}{l\,{\rm{d}}t})={\bf{0.008}}{{\boldsymbol{h}}}^{-{\bf{1}}}\\ \varepsilon  & = & 61MPa\\ \varphi  & = & (\frac{{\boldsymbol{\varepsilon }}{\boldsymbol{\varphi }}\,}{{\boldsymbol{\varepsilon }}})=(\frac{10.3\,{h}^{-1}}{61\,MPa\,})=0.1688\,MP{a}^{-1}{h}^{-1}\\ {{\boldsymbol{v}}}_{{\bf{sC}}} & = & (\frac{{\boldsymbol{\varepsilon }}{\boldsymbol{\varphi }}}{{{\boldsymbol{\Pi }}}_{{\bf{pe}}}})=(\frac{(61\,MPa\,)(0.17MP{a}^{-1}{h}^{-1})}{1524})\cong {\bf{0.007}}{{\boldsymbol{h}}}^{-{\bf{1}}}\end{array}$$

The data for *intact* stage IV sporangiophores were taken from Ortega *et al*.^4^ and Ortega^6^.

$$\begin{array}{rcl}l & = & 30\,mm=3\times {10}^{4}\,\mu m\\ \frac{{\rm{d}}l}{{\rm{d}}t} & = & 34.1\pm 3.1\,({\rm{SE}})\,\mu m\,mi{n}^{-1}=2046\pm 186\,({\rm{SE}})\,\mu m\,{h}^{-1}(n=20)\\ {{\boldsymbol{v}}}_{{\bf{sM}}} & = & (\frac{{\bf{d}}{\boldsymbol{l}}}{{\boldsymbol{l}}\,{\bf{d}}{\boldsymbol{t}}})={\bf{0.068}}\pm {\bf{0.006}}\,({\bf{SE}})\,{{\boldsymbol{h}}}^{-{\bf{1}}}({\bf{n}}={\bf{20}})\\ {\boldsymbol{\varepsilon }} & = & {\bf{60.9}}\pm {\bf{5.1}}\,({\bf{SE}})\,{\boldsymbol{MPa}}\,({\boldsymbol{n}}={\bf{27}})\\ \varphi ({\rm{largest}}) & = & (\frac{m}{l})=(\frac{997+160\,\mu m\,mi{n}^{-1}MP{a}^{-1}}{3.0\times {10}^{4}\,\mu m})\\  & = & 0.039\,mi{n}^{-1}MP{a}^{-1}=2.3\,{h}^{-1}MP{a}^{-1}\\ \varphi ({\rm{smallest}}) & = & (\frac{m}{l})=(\frac{997-160\,\mu m\,mi{n}^{-1}MP{a}^{-1}}{\,\,3.0\times {10}^{4}\,\mu m})\\  & = & 0.028\,mi{n}^{-1}MP{a}^{-1}=1.7\,{h}^{-1}MP{a}^{-1}\\ {{\boldsymbol{v}}}_{{\bf{sC}}}({\rm{largest}}) & = & (\frac{{\boldsymbol{\varepsilon }}{\boldsymbol{\varphi }}\,}{{{\boldsymbol{\Pi }}}_{{\bf{pe}}}})\,=(\frac{(66\,MPa)(2.3\,MP{a}^{-1}{h}^{-1})}{1524})\cong {\bf{0.099}}{{\boldsymbol{h}}}^{-{\bf{1}}}\\ {{\boldsymbol{v}}}_{{\bf{sC}}}({\rm{smallest}}) & = & (\frac{{\boldsymbol{\varepsilon }}{\boldsymbol{\varphi }}\,}{{{\boldsymbol{\Pi }}}_{{\bf{pe}}}})=(\frac{(55.8\,MPa\,)(1.7\,MP{a}^{-1}{h}^{-1})}{1524})\cong {\bf{0.062}}{{\boldsymbol{h}}}^{-{\bf{1}}}\\ {{\boldsymbol{v}}}_{{\bf{sC}}} & = & {\bf{0.0805}}\pm {\bf{0.0185}}{{\boldsymbol{h}}}^{-{\bf{1}}}\end{array}$$

The data for intact stage I sporangiophores were taken from Ortega^6^.

$$\begin{array}{rcl}l & = & 20\,mm=2\times {10}^{4}\mu m\\ \frac{{\rm{d}}l}{{\rm{d}}t} & = & 7.1\pm 0.7\,({\rm{SE}})\,\mu m\,mi{n}^{-1}=426\pm 42\,({\rm{SE}})\,\mu m\,{h}^{-1}({\rm{n}}=20)\\ {{\boldsymbol{v}}}_{{\bf{sM}}} & = & (\frac{{\bf{d}}{\boldsymbol{l}}}{{\boldsymbol{l}}\,{\bf{d}}{\boldsymbol{t}}})={\bf{0.021}}\pm {\bf{0.002}}\,({\bf{SE}})\,{{\boldsymbol{h}}}^{-{\bf{1}}}({\bf{n}}={\bf{17}})\\ {\boldsymbol{\varepsilon }} & = & {\bf{68.9}}\pm {\bf{5.6}}\,({\bf{SE}})\,{\boldsymbol{MPa}}({\boldsymbol{n}}={\bf{27}})\\ \varphi  & = & (\frac{{\rm{m}}}{l})=(\frac{116\,\mu m\,mi{n}^{-1}MP{a}^{-1}}{2\times {10}^{4}\,\mu m})(60\,{\rm{\min }}\,{h}^{-1})=0.348MP{a}^{-1}{h}^{-1}\\ \varphi ({\rm{largest}}) & = & (\frac{m}{l})=(\frac{116+18.8\,\mu m\,mi{n}^{-1}MP{a}^{-1}}{2.0\times {10}^{4}\mu m})\\  & = & 0.00674\,mi{n}^{-1}MP{a}^{-1}=0.4044\,{h}^{-1}MP{a}^{-1}\\ \varphi ({\rm{smallest}}) & = & (\frac{m}{l})=(\frac{116-18.8\,\mu m\,mi{n}^{-1}MP{a}^{-1}}{2.0\times {10}^{4}\,\mu m})\\  & = & 0.00486\,mi{n}^{-1}MP{a}^{-1}=0.29\,{h}^{-1}MP{a}^{-1}\\ {{\boldsymbol{v}}}_{{\bf{sC}}}({\rm{largest}}) & = & (\frac{{\boldsymbol{\varepsilon }}{\boldsymbol{\varphi }}}{{{\boldsymbol{\Pi }}}_{{\bf{pe}}}})=(\frac{(74.5\,MPa)(0.40\,MP{a}^{-1}{h}^{-1})}{1524})\cong {\bf{0.020}}{{\boldsymbol{h}}}^{-{\bf{1}}}\\ {{\boldsymbol{v}}}_{{\bf{sC}}}({\rm{smallest}}) & = & (\frac{{\boldsymbol{\varepsilon }}{\boldsymbol{\varphi }}}{{{\boldsymbol{\Pi }}}_{{\bf{pe}}}})=(\frac{(63.3\,MPa)(0.29\,MP{a}^{-1}{h}^{-1})}{1524})\cong {\bf{0.012}}{{\boldsymbol{h}}}^{-{\bf{1}}}\\ {{\boldsymbol{v}}}_{{\bf{sC}}} & = & {\bf{0.016}}\pm {\bf{0.004}}{{\boldsymbol{h}}}^{-{\bf{1}}}\end{array}$$

### Constant-tension extension experiment - load removed

Unidirectional constant-tension extension experiments were conducted on frozen and then thawed walls of the stage IV sporangiophore using the same method and experimental apparatus used and described by Ortega *et al*.^30^ (for Fig. 4). A five-millimeter long section of the wall from a stage IV sporangiophore, that includes the growth zone, was adapted to a bathing solution of pure water for 20 min in a test apparatus. Then a tensile load of 1.24 gram was applied to the wall. The tensile load produces longitudinal stress and longitudinal extension of the wall (810 μm). After the extension, the length remained constant for four minutes (11–15 min). At 15 min (marked by the downward pointing arrow), 50 mM potassium-acetate buffer, pH 4.5, replaces the bathing water in the test apparatus. It can be seen that “creep” extension begins immediately and continues for seven minutes. The creep extension behavior produced by the 50 mM potassium-acetate buffer, pH 4.5, is very similar to that obtained when the pH of the solution was lower to 4.6 by adding a predetermined amount of pH Red 4.0 buffer to the bathing water^30^. Afterwards (22–25 min), the wall section length remains constant for three minutes. At 25 min, the tensile load of 1.24 grams (*σ* = 42 MPa; see Ortega *et al*.^30^ for detailed stress calculations) is slowly removed and the wall section decreases in length over the next three minutes. The decrease in length is “recovered” elastic extension.

The data obtained from this experiment is used for the following calculations. When *σ* is constant (11–25 min) the change in length (creep) stimulated by the decrease in pH is governed by; *v*_s_ = (d*l*/d*t*)/*l* = *ϕ* (*σ* − *σ*_C_), and *ϕ* = *v*_s_/(*σ* − *σ*_C_). When the stress is removed (at 25 min), then the decrease in length is governed by; −(d*l*/d*t*)/*l* = −(1/*ε*) d*σ*/d*t*, and *ε* = Δ*σ*/(Δ*l*/*l*). From the experiment and Fig. 4 we get:

$$\begin{array}{ccc}l & = & 5.0\,{\rm{m}}{\rm{m}}=5000\,\mu {\rm{m}}={\rm{l}}{\rm{e}}{\rm{n}}{\rm{g}}{\rm{t}}{\rm{h}}\,{\rm{o}}{\rm{f}}\,{\rm{c}}{\rm{e}}{\rm{l}}{\rm{l}}\,{\rm{w}}{\rm{a}}{\rm{l}}{\rm{l}}\,{\rm{b}}{\rm{e}}{\rm{i}}{\rm{n}}{\rm{g}}\,{\rm{t}}{\rm{e}}{\rm{s}}{\rm{t}}{\rm{e}}{\rm{d}}\\ dl/dt & = & 17\,\mu {\rm{m}}\,{min}^{-1}={\rm{a}}{\rm{v}}{\rm{e}}{\rm{r}}{\rm{a}}{\rm{g}}{\rm{e}}\,{\rm{c}}{\rm{r}}{\rm{e}}{\rm{e}}{\rm{p}}\,{\rm{e}}{\rm{x}}{\rm{t}}{\rm{e}}{\rm{n}}{\rm{s}}{\rm{i}}{\rm{o}}{\rm{n}}\,{\rm{r}}{\rm{a}}{\rm{t}}{\rm{e}}\,{\rm{d}}{\rm{u}}{\rm{r}}{\rm{i}}{\rm{n}}{\rm{g}}\,7\,min\,{\rm{i}}{\rm{n}}{\rm{t}}{\rm{e}}{\rm{r}}{\rm{v}}{\rm{a}}{\rm{l}}\\ {v}_{{\rm{s}}} & = & (dl/dt)/l=17\,\mu {\rm{m}}\,{min}^{-1}/5000\,\mu {\rm{m}}=0.0034\,{min}^{-1}\\ \sigma -{\sigma }_{{\rm{C}}} & = & 42\,{\rm{M}}{\rm{P}}{\rm{a}}-16.3\,{\rm{M}}{\rm{P}}{\rm{a}}=25.7\,{\rm{M}}{\rm{P}}{\rm{a}}=effective\,{\rm{s}}{\rm{t}}{\rm{r}}{\rm{e}}{\rm{s}}{\rm{s}}\,{\rm{d}}{\rm{u}}{\rm{r}}{\rm{i}}{\rm{n}}{\rm{g}}\,{\rm{c}}{\rm{r}}{\rm{e}}{\rm{e}}{\rm{p}}\,{\rm{e}}{\rm{x}}{\rm{t}}{\rm{e}}{\rm{n}}{\rm{s}}{\rm{i}}{\rm{o}}{\rm{n}}\\ \varphi  & = & {v}_{{\rm{s}}}/(\sigma -{\sigma }_{{\rm{C}}})=1.3\times {10}^{-4}\,{min}^{-1}{{\rm{M}}{\rm{P}}{\rm{a}}}^{-1}\\ \varepsilon  & = & {\rm{\Delta }}\sigma /({\rm{\Delta }}l/l)=(\mathrm{-42}\,{\rm{M}}{\rm{P}}{\rm{a}})/(\mathrm{-62}\,\mu m/5000\,\mu m)=3387\,{\rm{M}}{\rm{P}}{\rm{a}}\end{array}$$

$${{\boldsymbol{\Pi }}}_{{\bf{pe}}}=\frac{{\boldsymbol{\varepsilon }}{\boldsymbol{\varphi }}}{{{\boldsymbol{v}}}_{{\bf{s}}}}\approx {\bf{130}}$$

## Electronic supplementary material

Supplementary Information
