# Supplementary material for: Dimensionless number is central to stress relaxation and expansive growth of the cell wall
Source: Sci Rep. 2017 Jun 7;7:3016. doi: 10.1038/s41598-017-03002-6 (PMC5462804; doi:10.1038/s41598-017-03002-6)
Supplement: Supplementary file 1 — Supplementary Information [file 41598_2017_3002_MOESM1_ESM.pdf]

Glossary

A - area

l – length of the cell

L_p – hydraulic conductivity of the plasma (cell) membrane

L – relative hydraulic conductance of the plasma membrane = (L_p A / V)

dL/dt – elongation rate

P – turgor pressure, the difference in pressure inside and outside the cell

P^* – dimensionless turgor pressure

P_C – critical turgor pressure

P_i – initial value of the turgor pressure at the beginning of a stress relaxation experiment

dP/dt – rate of change of turgor pressure

RH – relative humidity

t – time

t_c – time constant of the exponential decay

$T_{1/2}$ – halftime of the exponential decay

V – volume

V_{cw} – volume of the cell wall chamber

v_{cw} = (dV/dt)/V – relative rate of change cell wall chamber volume

v_{cw}^* – dimensionless v_{cw}

v_s = v_{cw} = constant; a constant average value over some time interval

v_{sC} = calculated value of v_s

v_{sM} = measured value of v_s

v_w^* – dimensionless v_w (relative rate of change in water volume)

v_T^* – dimensionless v_T (relative rate of change in water volume lost through transpiration)

ε – volumetric elastic modulus

$\Delta\pi^*$ – dimensionless osmotic pressure difference across the plasma membrane

Π – dimensionless group of variables

$\Pi_{ev} = P_C / \varepsilon$

$\Pi_{pe} = \varepsilon \phi / v_s$

$\Pi_{pv} = \phi P_C / v_s$

ϕ – irreversible wall extensibility
